# Detection of *Leishmania* parasites in the testis of a dog affected by orchitis: case report

**DOI:** 10.1186/1756-3305-5-216

**Published:** 2012-09-28

**Authors:** Laura Manna, Orlando Paciello, Rossella Della Morte, Angelo Elio Gravino

**Affiliations:** 1 Department of Veterinary Clinical Science, University of Naples Federico II, Via F. Delpino 1, 80137, Naples, Italy; 2 Department of Animal Pathology and Health, University of Naples Federico II, Via F. Delpino 1, 80137, Naples, Italy; 3 Department of Biological Structures, Functions and Technologies, University of Naples Federico II, Via F. Delpino 1, 80137, Naples, Italy

**Keywords:** Dog, Leishmaniasis, Orchitis, Real-time PCR, Testis

## Abstract

**Background:**

Transmission of canine leishmaniasis (CanL), a severe infection caused by *L. infantum,* usually occurs through the sand fly bite to the vertebrate host. A venereal route of transmission has also been suggested, but this issue is still controversial.

**Findings:**

Here, we report a case of a dog affected by orchitis showing a clinical profile of *L. infantum* infection. By exploiting a real-time PCR assay, we detected a significantly higher DNA load of the parasite in the lymph node and testis than in blood and urine samples collected from the dog.

**Conclusions:**

Our results suggest that: 1) *L. infantum* infection can be associated with testicular lesions in naturally infected dogs; 2) genital involvement could result in shedding of the parasites in the semen, favoring venereal transmission of the disease.

## Findings

### Introduction

Canine leishmaniasis (CanL) is a severe sandfly-borne infection caused by the protozoan parasite *Leishmania infantum* (syn. *L*. *chagasi)* widely distributed in temperate and subtropical countries. The clinical manifestations of the disease range from unapparent subclinical infections to a systemic disease characterized by weight loss, lymphadenopathy, hemorrhagic diarrhea, ocular lesions, and hyperthermia, frequently associated also with dermatological lesions. Dogs are the main reservoir of *L. infantum* and they play a central role in the transmission of the disease to humans. The prevalence and the incidence of CanL have been underestimated until now [1,2]. Transmission of the disease usually occurs through the phlebotomine sand fly bite to the vertebrate host; however, CanL transmission through blood transfusion has been documented [3]. Recent evidence also suggests a venereal route of transmission due to the presence of parasites in the semen of infected dogs [4-7]. However, the *L. infantum* tropism to the canine genital system is still a controversial question. Here, we report a case of orchitis in a leishmaniotic dog associated with the presence of *Leishmania* DNA in the testis.

### Case report

**Figure 1:**
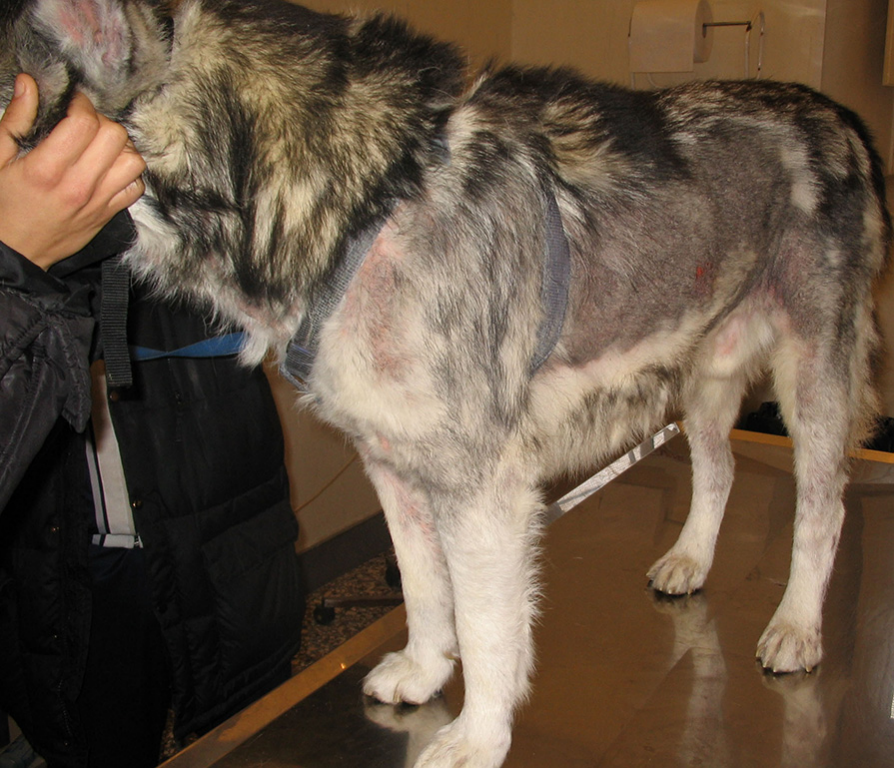
A 6-yr-old male Husky dog showing clear evidence of dermatitis.

**Figure 2:**
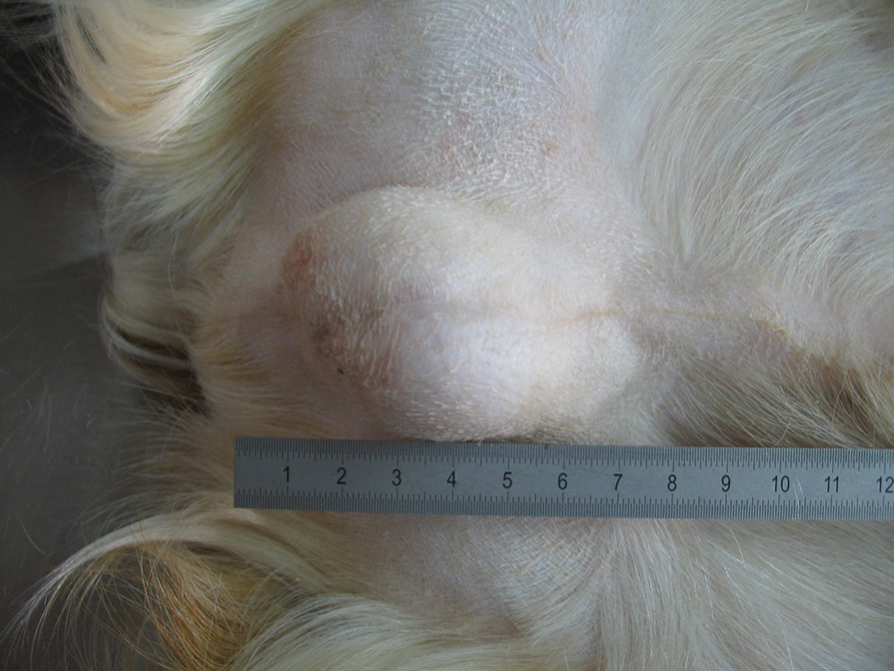
Evident enlargement of right testis in the Husky dog.

A 6-yr-old male Husky was hospitalized at the Department of Veterinary Clinical Science of the Federico II University of Naples for the evaluation of a seborrhoeic, pruriginous dermatitis (Figure 1) with a history of two months of alopecia and pruritus. The dog had been treated for sarcoptic mange without any improvement. Physical examination showed an enlargement of the right testis (Figure 2). No lymphadenopathy was recorded. Routine fine needle aspiration cytology from the enlarged testis was performed by the attending pathologist. Air-dried slides were prepared, and immediately stained with May Grunwald-Giemsa Quick Stain (Bio-Optica, Milan). Clinical laboratory parameters were evaluated. Indirect immunofluorescent antibody test (IFAT) for *Leishmania spp*. was carried out, and samples from blood, urine, lymph node, and testis were collected, and processed for the evaluation of *Leishmania* DNA load by real-time PCR assay as previously described [8]. 

Cytological examination of testis samples showed testicular round cells with course nuclear chromatin, single large, prominent nucleoli, and moderate amounts of basophilic cytoplasm. The presence of many inflammatory cells, such as neutrophils, with nuclear degenerative changes, several lymphocytes, plasma cells and a large number of macrophages containing intracellular *Leishmania* amastigotes was observed. A diagnosis of chronic orchitis due to *Leishmania* spp. was made. Haematological and biochemical parameters were consistent with CanL (Table 1) as demonstrated by the anemia, increased levels of total proteins, and high UP/C ratio. [9,10] Real time PCR and IFAT results confirmed the infection by *L. infantum* of the dog (Table 2). 

**Table 1:** Dog laboratory parameters recorded at time 0 (T0) and after 1 (T1), 2 (T2), 3 (T3), 6 (T6) and 12 (T12) months from the diagnosis

**Substrate**	**Parameter**	**T0**	**T1**	**T2**	**T3**	**T6**	**T12**	**Normal range**
**Serum**	Urea (mg/dl)	45	47	29	31	28	26	25-50
	Creatinine (mg/dl)	1.65	1.3	1.26	1.47	1.48	1.54	<1.8
	ALT (UI/L)	27	23	25	24	26	33	10-47
	Glucose (mg/dl)	81	80	83	81	76	86	60-110
	Total proteins (g/dl)	9.4	8.7	7.4	7.4	7.4	6.2	6-7.8
	Gamma globulin (g/dl)	3.63	2.84	2.16	2.16	2.16	2.84	0.9-2.2
	Beta globulin (g/dl)	2.39	2.43	2.31	0.31	0.31	0.34	0.6-1.4
	Alpha 2 globulin (g/dl)	0.41	1.55	1.11	1.11	1.11	0.82	0.3-1.1
	Alpha 1 glubulin (g/dl)	3.50	1.20	1.04	1.04	1.04	0.67	0.2-0.5
	Albumin (g/dl)	2.35	0.70	1.12	1.12	1.12	0.70	2.3-3.4
	A/G ratio	2.25	2.46	1.96	1.96	1.96	0.53	0.7-1.11
**Blood**	RBC (x 106/μL)	3.99	6.01	4.47	4.71	5.1	5.2	5.6-8.6
	Hb (g/dL)	9.9	11.3	11.6	12.2	13.5	16.3	13.5-18
	WBC (x 10 3/μL)	22.1	24.2	15.1	13.4	11.3	8	6-17
	PLT (x 103/μL)	456	366	607	370	343	310	200-500
**Urine**	Density (g/ml)	1031	1021	1021	1015	1010	1015	1015-1040
	UP/C ratio	5.5	2.4	2.3	0.3	0.4	0.1	<5

**Table 2:** ***Leishmania *load (parasite DNA/ml) in blood, lymph node aspirates, urine and testis samples at time 0 (T0) and after 1 (T1), 2 (T2), 3 (T3), 6 (T6) and 12 (T12) months from the diagnosis **

**Substrate**	**T0**	**T1**	**T2**	**T3**	**T6**	**T12**
**Blood**	321.91	205	13	10	11	4.05
**Lymph node**	23602.83	1694.78	1541.89	4.95	10	2.02
**Urine**	779	178	80	30	10	3.0
**Testicle**	14406.57	116.26	2.88	2.18	3.05	10.64
**IFAT**	1:1280	1:640	1:1280	1:80	1:160	1:320

The dog was treated with a combination of miltefosine [11] at a dose of 2 mg/kg/day per 28 days and allopurinol at a dose of 10 mg/kg/day for all the observation period (1 year). For follow-up assessment, biological samples were collected at the time of diagnosis (T0), and 1, 2, 3, 6, 12 months after the treatment had started (T1, T2, T3, T6 and T12, respectively).

Before therapy, a very high parasite DNA load was detected in lymph node aspirates (23602.83 parasite DNA/ml) and testis biopsy (14406.57 parasite DNA/ml), whereas lower load values were observed in blood and urine samples (321.91 and 779 parasite DNA/ml, respectively). These results confirmed previous data on the comparative analysis of different tissues for CanL diagnosis by conventional or real-time PCR [12]. After 1 month of therapy, a progressive clinical improvement was observed, including a moderate decrease of anti-*Leishmania* antibody titer together with a strong reduction of *Leishmania* DNA load in all biological samples. However, the complete elimination of *Leishmania* DNA was never observed in all tissues. After the therapy, at 1 month follow-up, the cytological evaluation of the testis did not show any inflammatory cell.

### Conclusions

This case report confirms that CanL can be associated with testis lesions (orchitis) in naturally infected dogs. The *Leishmania* amastigotes in the testis apparently act as a causing factor, triggering the inflammatory response. Experimental infection of hamsters with *L. donovani* resulted in testicular amyloidosis, degeneration, progressive atrophy, and azoospermy. In this experimental model, the degenerative changes were also associated with infiltration of lymphocytes and macrophages containing amastigotes in the testes [13]. In contrast, testicular amyloidosis was not observed in our case and in other studies [4]. In human visceral leishmaniasis, testicular involvement has not been extensively studied, but there is one study reporting amastigotes in macrophages obtained by fine needle aspiration of the testes from a boy with a severe lymphoblastic leukemia [14].

Genital involvement during visceral leishmaniosis could result in shedding of *Leishmania* in the semen, favoring venereal transmission of the disease, such as reported in humans [15]. Venereal and vertical transmission of *L. infantum* in naturally infected dogs in Germany has been reported [7]. Inflammation associated with erosions and/or ulcerations and the presence of amastigotes in the penis and prepuce, as well as the presence of macrophages containing amastigotes migrating through the urethral epithelium might contribute to *Leishmania* shedding in the semen. Although the biological vector is the most important route of transmission, the possibility of CanL venereal transmission has epidemiological significance, mostly in relation to the implementation of an eradication program. This route of transmission in dogs is particularly relevant in areas where treatment and vaccination of dogs against CanL are frequent, since under those conditions potentially infective dogs may not be readily identified, thus increasing the chance of using these dogs in reproduction, which may favor spread of the disease.

## Competing interests

The authors declare that they have no competing interests.

## Authors’ contributions

LM conceived the study, participated in the design, data collection, and analysis of real-time PCR study, and drafted the manuscript. OP performed cytological analysis of testis tissues. RDM participated in the hematological and biochemical data collection and analysis, and cooperated in drafting the manuscript. AEG participated in the overall study design and realization. All authors read and approved the final version of the manuscript.
